# Investigation of Grating-Assisted Trimodal Interferometer Biosensors Based on a Polymer Platform

**DOI:** 10.3390/s18051502

**Published:** 2018-05-10

**Authors:** Yuxin Liang, Mingshan Zhao, Zhenlin Wu, Geert Morthier

**Affiliations:** 1 Photonics Research Group, Department of Information and Technology, Ghent University, 9000 Ghent, Belgium; yuxin.liang@ugent.be; 2 School of Physics and Optoelectronic Engineering, Dalian University of Technology, Dalian116023, China; mszhao@dlut.edu.cn (M.Z.); zhenlinwu@dlut.edu.cn (Z.W.)

**Keywords:** trimodal waveguide, biosensors, long period grating, polymer waveguide

## Abstract

A grating-assisted trimodal interferometer biosensor is proposed and numerically analyzed. A long period grating coupler, for adjusting the power between the fundamental mode and the second higher order mode, is investigated, and is shown to act as a conventional directional coupler for adjusting the power between the two arms. The trimodal interferometer can achieve maximal fringe visibility when the powers of the two modes are adjusted to the same value by the grating coupler, which means that a better limit of detection can be expected. In addition, the second higher order mode typically has a larger evanescent tail than the first higher order mode in bimodal interferometers, resulting in a higher sensitivity of the trimodal interferometer. The influence of fabrication tolerances on the performance of the designed interferometer is also investigated. The power difference between the two modes shows inertia to the fill factor of the grating, but high sensitivity to the modulation depth. Finally, a 2050 2π/RIU (refractive index unit) sensitivity and 43 dB extinction ratio of the output power are achieved.

## 1. Introduction

Integrated photonic biosensors have demonstrated their great potential for clinical, chemical and biological diagnostics in the past years. They have many advantages, such as high sensitivity, compact dimensions, mechanical stability, and immunity to electromagnetic interference [1,2]. Moreover, they are suitable for point-of-care systems, as they can realize label-free, real-time monitoring, and they can be integrated into a lab-on-a-chip. Many structures have been demonstrated such as Mach–Zehnder interferometers (MZIs) [3], microring resonators [4,5,6], photonic crystals [7,8], and plasmonic structures [9,10]. Among them, the MZI biosensor is one of the most attractive structures, because it is simple and easy in design, fabrication, and measurement. Meanwhile, it can achieve a high sensitivity and a small limit of detection (LOD) [2]. 

A conventional MZI biosensor is composed of two arms: a sensing arm and a reference arm. Enough spatial separation between the two arms is required to prevent evanescent coupling. In order to improve the performance, MZI biosensors have been demonstrated on various waveguides, such as strip and rib waveguides [11], arrow waveguides [12], plasmonic waveguides [13], and slot waveguides [14]. A novel structure, a bimodal interferometer, was proposed and demonstrated in 2011 by the group of Prof. Lechuga [15]. Unlike the interference between the two different arms of the conventional MZI structures, the interference is obtained between the fundamental mode and the vertical first higher order mode in the bimodal interferometer. As the environmental changes can affect the effective indices of the two modes differently, there will be a phase difference after propagation through the sensing region which modulates the output power. However, the fabrication process of the bimodal vertical waveguide is quite complicated, as two steps of lithography and etching are required to fabricate the discontinuous junction. In addition, the input power is split equally between the two arms in a conventional MZI, in order to give maximal fringe visibility, which influences the LOD. However, the discontinuous part in a bimodal interferometer cannot act as a 3 dB splitter. In order to solve this problem, the grating assisted bimodal waveguide was proposed [16].

In order to simplify the fabrication process, a lateral bimodal waveguide interferometer was proposed and demonstrated [17]. As it has the same height everywhere, the two steps of lithography and etching are avoided, which makes the fabrication much simpler. Furthermore, the coupling ratio between the two lateral modes and the input light is adjusted by changing the position of the single mode waveguide. However, there is a large insertion loss as the input single mode waveguide is located away from the bimodal waveguide center to provide the same power to the two lateral modes. In 2015, a trimodal waveguide was proposed, which makes use of the interference between the fundamental mode and the second higher order mode. As the second higher order mode has a larger evanescent tail than the first higher order mode, it can reach a higher sensitivity [18]. However, if the single mode waveguide is not in the middle of the trimodal waveguide, the first higher order mode will be excited, which will affect the interference between the fundamental mode and the second higher order mode. As a result, the coupling efficiency between the two modes and the input light cannot be adjusted anymore by changing the position of the input waveguide. The suffered fringe visibility will influence the LOD.

In this article, we propose a modified trimodal waveguide interferometric biosensor. The new trimodal interferometer utilizes long period gratings (LPGs) as the couplers at input and output ports. The LPG couplers can adjust the power ratio between the fundamental mode and the second higher order mode without exciting the first higher order mode. When the 3 dB splitter and the combiner have been optimized by changing the LPG parameters, a maximal fringe visibility is achieved without extra insertion loss. The extinction ratio of the output power can be improved by 36 dB compared to that of the conventional trimodal interferometer, which results in a better LOD. The design is based on a polymer (ma-P 1205 from Micro Resist Technology) which can provide cost effectiveness and mass production at the same time. Moreover, the polymer is easily functionalized, which is necessary for biosensors [19].

## 2. Principle of LPG Assisted Trimodal Interferometer

**Figure 1:**
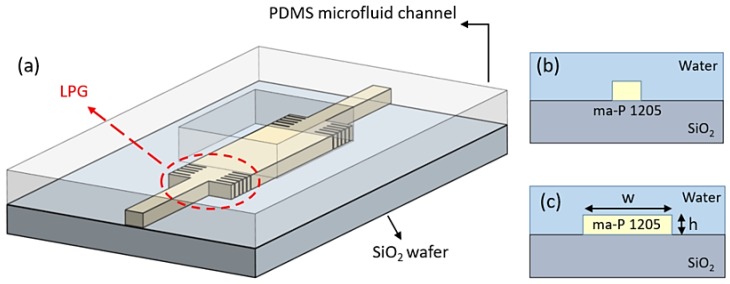
(**a**) Schematic of trimodal interferometer; (**b**) Input single waveguide cross-section; (**c**) Trimodal waveguide cross-section.

The schematic of the trimodal interferometer is shown in Figure 1a. The interferometer is composed of single mode waveguides as in/output ports, a trimodal waveguide for sensing, and LPGs as splitter/combiner. Only transverse electric (TE) polarization is considered for the sake of simplicity, as the transverse magnetic (TM) polarization works similarly. A polymer (ma-P 1205) is chosen as the core layer, which is transparent at both the visible and infrared wavelengths. The under cladding layer is SiO_2_, and the upper cladding layer is assumed to be water, which is usually used as solvent in biosensing. All the simulations are executed near the wavelength of 633 nm as the water absorption at this wavelength is much lower than at the infrared wavelength. The refractive index of ma-P 1205 is 1.644 at this wavelength. As the higher order mode is supported laterally, the trimodal interferometer has the same thickness everywhere, which simplifies the fabrication process. Moreover, as the higher mode distributes laterally, the interaction between the light and the analyte on the top surface has been enhanced, which makes it suitable for specific detection. The cross-sections of the single waveguide and the trimodal waveguide are also illustrated in Figure 1b,c, respectively.

The trimodal interferometer can act as a normal MZI interferometer. For conventional trimodal interferometer, light is launched into the input waveguide and the power is coupled to the $E_{11}^{x}$ mode and the $E_{13}^{x}$ mode at the discontinuous junction. As the input waveguide is in the middle of the trimodal waveguide, no $E_{12}^{x}$ mode is excited. The interference pattern is from the $E_{11}^{x}$ mode and the $E_{13}^{x}$ mode in the same pathway, instead of two different physical pathways. As the two internal modes have different responses from the environment changing, a phase shift happens between the two modes when the environment refractive index changes, which finally modulates the output power. It is well known that the output power reaches maximal fringe visibility, which influences the LOD when different modes have the same power. After the sensing area, the interference happens at the second discontinuous junction. However, just about 10% power is coupled into the $E_{13}^{x}$ mode for normal trimodal interferometer, which results in bad fringe visibility and LOD. In addition, the power coupled to the $E_{13}^{x}$ mode cannot be adjusted by controlling the position of the input waveguide as bimodal interferometer does, because the $E_{12}^{x}$ mode will be excited when the input waveguide is not at the middle. In this article, LPG has been designed to adjust the power of different modes. As shown in Figure 1a, light is launched into the input waveguide and split equally into the $E_{11}^{x}$ mode and the $E_{13}^{x}$ mode by the discontinuous junction and the LPG splitter, resulting in maximal fringe visibility. After propagating through the sensing area, a phase difference will exist between the two modes, which finally modulates the output power. The LPG combiner and the second discontinuous junction intermix the two modes and couple the light to the single mode waveguide.

## 3. Sensitivity of the Trimodal Interferometer

**Figure 2:**
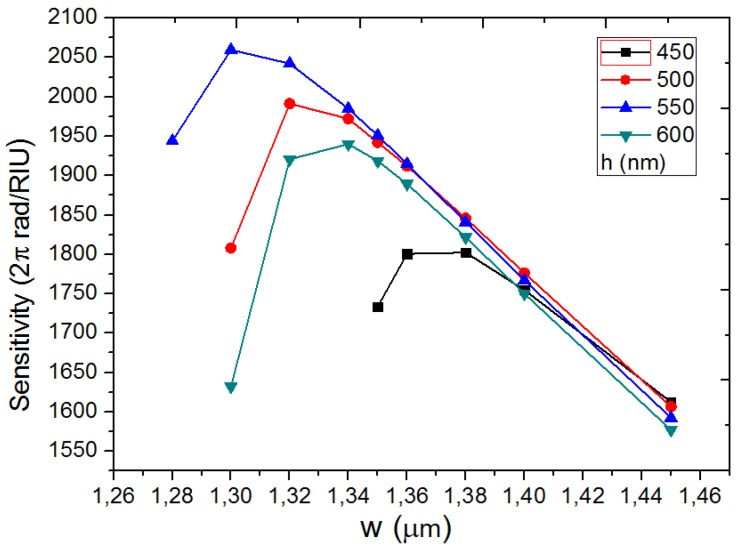
The relationship between sensitivity and waveguide width, *w* under different waveguide height, *h*.

The sensitivity of Interferometer structures is usually defined by the phase difference between the two arms (two different modes here) generated per RIU change of the environment outside the waveguide, i.e.,

(1) $$\frac{\partial \phi }{\partial n_{cl}}=2\pi \frac{L_{sensing}}{\lambda }\frac{\partial \left(n_{eff}^{0}-n_{eff}^{2}\;\right)}{\partial n_{cl}}$$

where $n_{eff}^{0}$ and $n_{eff}^{2}$ are the mode effective indices of the $E_{11}^{x}$ mode and $E_{13}^{x}$ mode and they are calculated at different $n_{cl}$ through mode solution with a finite difference mode solver (Mode Solution package from Lumerical Solutions, Inc., Vancouver, BC, Canada). After linear fitting, $\partial n_{eff}^{0}/\partial n_{cl}$ and $\partial n_{eff}^{2}/\partial n_{cl}$ can be obtained. $L_{sensing}$ is the total length of the sensing region, λ is the free space wavelength, and $n_{cl}$ is the refractive index of the environment. The sensitivity is determined by the waveguide structure. The relationship between sensitivity and waveguide width, *w*, under different waveguide height, *h*, is calculated and shown in Figure 2.

The sensing area length is considered as 15 mm, which is usually adopted in polymer-based interferometer biosensors. It can be seen that the sensitivity will increase and then decrease with increasing *w*. There is an optimal sensitivity for each *h*. It reaches the maximal value, 2050 2π/RIU, when *w* = 1.30 µm and *h* = 550 nm, which are adopted in the further simulations. It is much larger than for the bimodal interferometer reported in [17,18], which is due to larger evanescent tail of the second higher order mode. It enhances the interaction between the light and the environment.

## 4. Design of the LPG Coupler

**Figure 3:**
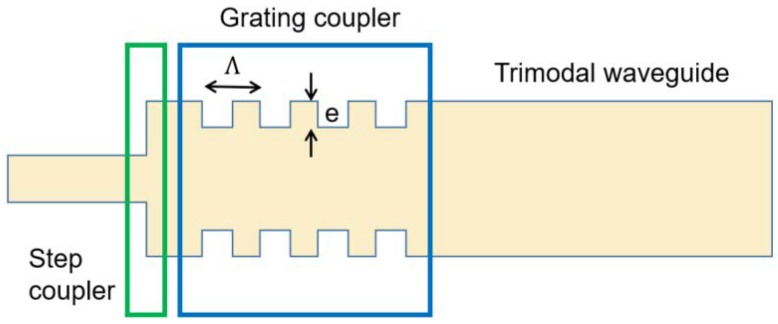
Schematic of a long period grating coupler.

**Figure 4:**
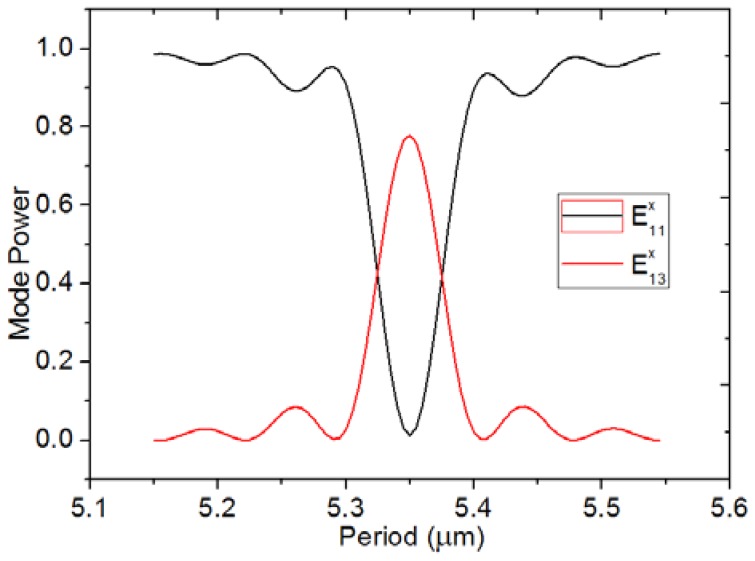
Change in the power of the fundamental mode and the first higher mode with the grating period with the period number being equal to 71.

The schematic of the LPG coupler is shown in Figure 3. Light from the input waveguide will first pass through the discontinuous junction, which is the same as the coupler in the normal trimodal interferometer. The following LPG can further adjust the power of the different two modes. The use of an LPG has been a well-known way to excite higher order modes in fiber optics. The higher order mode can be excited when the period of the grating, *Λ* is chosen to bridge the gap between the wave vectors of the fundamental and the higher order mode, i.e.,

(2) $$\Lambda =\frac{\lambda }{n_{eff}^{0}-n_{eff}^{2}}$$

where λ is the free space wavelength, and the grating introduces coupling between the $E_{11}^{x}$ mode and the $E_{13}^{x}$ mode. The coupling efficiency, κ, is determined by the parameters of the LPG: period (*Λ*), modulation depth (*e*), number of periods (*n_p_*), and fill factor (pitch/period). *Λ* is calculated as 5.4 μm from Formula (1), as $n_{eff}^{0}$ and $n_{eff}^{2}$ can be obtained by Mode Solution software, as mentioned above. As the calculated *Λ* is not that accurate from the formula, an optimization using a bidirectional eigenmode expansion solver (Mode Solution package from Lumerical Solutions, Inc., Vancouver, BC, Canada) is carried out. Figure 4 illustrates the relationship between the $E_{11}^{x}$ and $E_{13}^{x}$ power and *Λ*. Almost all the power is coupled to $E_{13}^{x}$ from $E_{11}^{x}$, when *Λ* is equal to 5.34 μm. The simulation is carried out at *n_p_* = 71.

**Figure 5:**
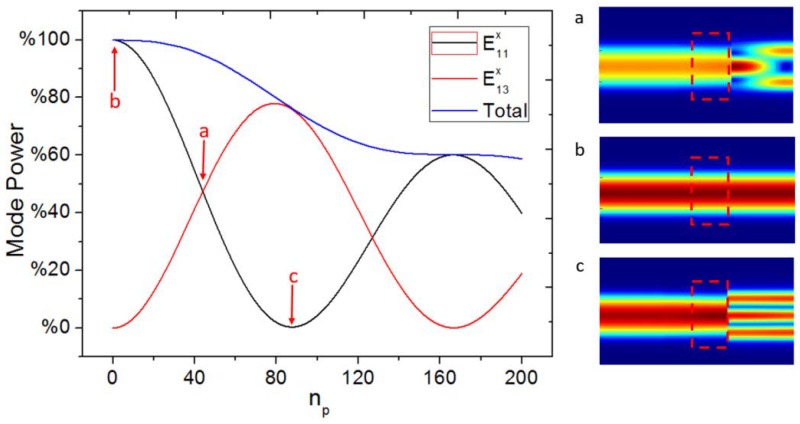
Variation of the fundamental mode and the second higher mode power with number of periods and propagating field pictures for (**a**) the $E_{11}^{x}$ mode and the $E_{13}^{x}$ mode having the same power, (**b**) just $E_{11}^{x}$ mode propagating in the trimodal waveguide, and (**c**) just $E_{13}^{x}$ mode propagating in the trimodal waveguide.

The length of the LPG or the number of periods (*n_p_*) also contribute to the coupling efficiency between the $E_{11}^{x}$ mode and the $E_{13}^{x}$ mode. The relationship between the power of the two modes and *n_p_* is shown in Figure 5. The coupling efficiency between the $E_{11}^{x}$ mode and the $E_{13}^{x}$ mode has been calculated without considering the input single mode waveguide. The input mode is chosen as the $E_{11}^{x}$ mode. The power of both modes fluctuates sinusoidally with *n_p_*, and the LPG acts similarly as a directional coupler. One difference is that the coupling efficiency can be adjusted by the grating length or the number of periods in an LPG. Another is that the total power decreases with increasing *n_p_*, due to the excitement of some radiation modes. It can be seen that both of the modes have the same power at *n_p_* = 44. Figure 5a illustrates the propagating fields for the $E_{11}^{x}$ mode and the $E_{13}^{x}$ mode propagating in the trimodal waveguide with the same power at *n_p_* = 44. Figure 5b illustrates the propagating field for only the $E_{11}^{x}$ mode propagating at *n_p_* = 0 as no power is coupled to the $E_{13}^{x}$ mode. Figure 5c illustrates the propagating field for only the $E_{13}^{x}$ mode propagating in the trimodal waveguide at *n_p_* = 88. From Figure 5c, it can be seen that the fundamental mode power has been coupled completely to the $E_{13}^{x}$ mode. As the simulations used a bidirectional eigenmode expansion solver (EME), just one period of the grating structure needs to be drawn in the simulation model (the part in the red rectangle). The grating part can be simulated through repeatedly calculating this period. 

Before the light enters into the LPG coupler, it has already been coupled to the two modes to a certain degree by the discontinuous junction. The design of the LPG should take this initial power ratio into account. At the discontinuous junction, the powers coupled to the $E_{11}^{x}$ mode and the $E_{13}^{x}$ mode are 79% and 11%, respectively, while 10% of the input power dissipates at the discontinuous junction. In order to achieve a 3 dB splitter, the number of periods is estimated at *n_p_* = 26. At this point, the $E_{11}^{x}$ mode and the $E_{13}^{x}$ mode get 42% and 43% of the power, respectively. The critical equality is not achieved as the number of periods must be an integer. 

## 5. Performance and Fabrication Tolerance Analysis

After propagating through the sensing region, the two modes will interfere with each other at the same LPG and discontinuous junction and the light is then exported to the output waveguide. The output power will be modulated by the phase difference between the two modes resulting from the sensing part. The output power can be expressed as

(3) $$I_{out}=\frac{1}{2}\left(I_{0}+I_{2}+2\sqrt{I_{0}I_{2}}\cos\left(\Delta \varphi \right)\right)$$

where $I_{0}$, $I_{2}$ and $I_{out}$ are the power of the $E_{11}^{x}$ mode, and the $E_{13}^{x}$ mode and the output power, respectively. *∆φ* is the phase difference between the two incident modes after propagating through the sensing area. The spectrum will suffer if the powers of the two different modes do not carry the same power, especially for the conventional trimodal interferometer as just 11% power is coupled into the $E_{13}^{x}$ mode. The bad fringe visibility will affect the LOD.

**Figure 6:**
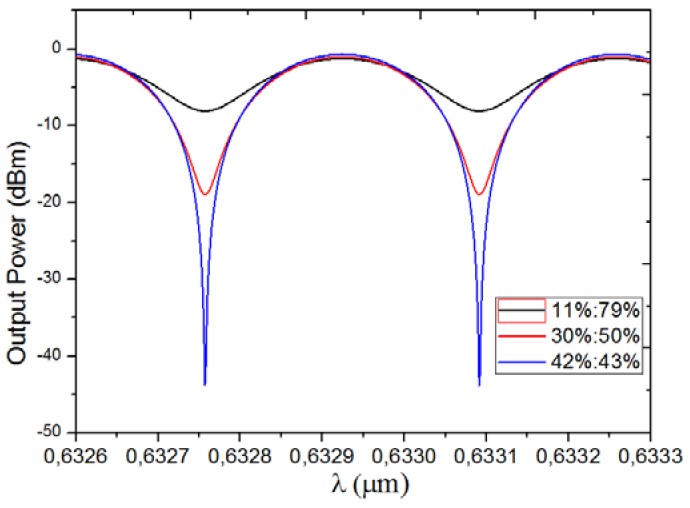
Output power spectrum for different power ratios.

The output power spectrum under different power ratios of the incident modes are shown in Figure 6. dBm is adopted as the unit of the output power, which is usually used in the detection, and the input power is assumed as 0 dBm. The extinction ratio of the output power for the conventional trimodal interferometer with the power ratio, 11%/79%, is about 7 dB, while it is about 43 dB for the modified trimodal interferometer in this work with the power ratio, 42%/43%, which is achieved by the optimized LPG from chapter 4. It is well known that the fringe visibility or the extinction ratio influences the LOD, as a high extinction ratio can suppress the influence of noise [18]. Normally the LOD deterioration influenced by bad fringe visibility, or the extinction ratio can be neglected when it is larger than 15 dB [20]. The output power spectrum for the power ratio, 30%/50%, is also illustrated in Figure 6, and gives a 20 dB extinction ratio.

**Figure 7:**
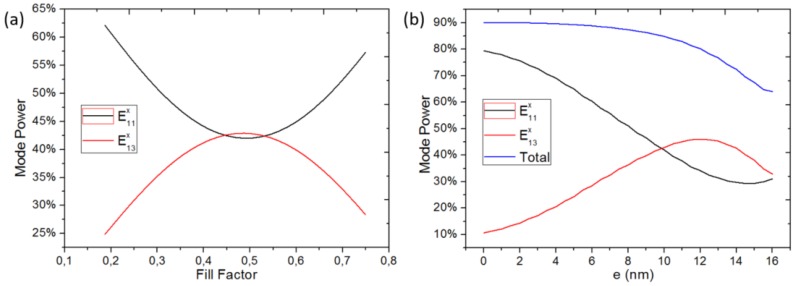
Change in the mode power with increasing fill factor (**a**) and modulation depth; *e* (**b**).

The influence of fabrication tolerances on the performance of LPGs has been investigated as well. Normally, polymer waveguide is fabricated by UV lithography or nanoimprinting, and the fabrication tolerance on the period is negligible. Therefore, only the tolerance on the fill factor and the modulation depth are investigated. Figure 7a,b illustrate the different mode powers as function of fill factor and modulation depth.

It can be seen that the mode powers are insensitive to the fill factor. The difference between the two mode powers stays within a 20% deviation, which gives a good enough fringe visibility for a fill factor between 0.28 and 0.70 and means that the wider part of the grating can change in length from 1.5 μm to 3.7 μm. However, the powers of the two modes are more sensitive to the modulation depth. Moreover, the insertion loss is also affected by modulation depth. In order to keep the power difference of the two modes within 20% and the output power larger than 70%, the modulation depth should be 10±3 nm. The fringe visibility of the entire splitter composed of the discontinuous junction and the LPG was also calculated versus the wavelength. The difference between the mode powers stays within 20% for a 20 nm wavelength window, which is enough for the biosensing.

## 6. Conclusions

A novel trimodal waveguide interferometric biosensor has been numerically demonstrated. It is more sensitive than a conventional bimodal interferometer biosensor, as the second higher order mode has deeper evanescent wave. For interferometric sensors, the two arms (two different modes in this article) should have the same optical power, in order to get maximal fringe visibility. In a lateral bimodal interferometer, the mode power ratio can be adjusted by controlling the location of the single mode input waveguide to get a maximal fringe visibility. However, this method cannot be applied for a trimodal waveguide interferometer, as the first higher order mode will be excited as well when the input waveguide is not in the middle. Moreover, this method also brings large insertion loss. A long period grating coupler has been investigated as an alternative. It can adjust the power of the fundamental mode and the second higher order mode without exciting the first higher order mode with a low insertion loss. It works similarly to the directional coupler, which can adjust the power in the two arms. Using such a long period grating, the interferometer can achieve maximal fringe visibility by adjusting the power of the two modes, which can improve the LOD. The fabrication tolerances were also studied. The power difference between the two modes is insensitive to the fill factor and sensitive to the modulation depth. On the other hand, the power difference can stay within 20%, which allows a good fringe visibility for a 20 nm wavelength window. Finally, the sensitivity reaches 2050 2π/RIU for a 15 mm sensing region. It is much larger than for bimodal interferometer biosensors which have been reported. A 43 dB extinction ratio is achieved, which is 36 dB larger than for a conventional trimodal interferometer, and a better LOD can be expected.

## References

[1] Fan X., White I.M., Shopova S.I., Zhu H., Suter J.D., Sun Y. (2008). Sensitive optical biosensors for unlabeled targets: A review. Anal. Chim. Acta.

[2] Estevez M.C., Alvarez M., Lechuga L.M. (2012). Integrated optical devices for lab-on-a-chip biosensing applications. Laser Photonics Rev..

[3] Luff B.J., Wilkinson J.S., Piehler J., Hollenbach U., Ingenhoff J., Fabricius N. (1998). Integrated Optical Mach-Zehnder Biosensor. J. Lightw. Technol..

[4] Vos K.D., Bartolozzi I., Schacht E., Bienstman P., Baets R. (2007). Silicon-on-Insulator microring resonator for sensitive and label-free biosensing. Opt. Express.

[5] Wang L., Ren J., Han X., Claes T., Jian X., Bienstman P., Baets R., Zhao M., Morthier G. (2012). A Label-Free Optical Biosensor Built on a Low-Cost Polymer Platform. IEEE Photonics J..

[6] Morarescu R., Pal P., Beneitez N., Missinne J., Steenberge G., Bienstman P., Morthier G. (2016). Fabrication and Characterization of High-Optical-Quality-Factor Hybrid Polymer Microring Resonators Operating at Very Near Infrared Wavelengths. IEEE Photonics J..

[7] Jahns S., Bräu M., Meyer B.-O., Karrock T., Gutekunst S.B., Blohm L., Selhuber-Unkel C., Buhmann R., Nazirizadeh Y., Gerken M. (2015). Handheld imaging photonic crystal biosensor for multiplexed, label-free protein detection. Biomed. Opt. Express.

[8] Feng S., Jiang J.-H., Rashid A.A., John S. (2016). Biosensor architecture for enhanced disease diagnostics: Lab-in-a-photonic-crystal. Opt. Express.

[9] Gao Y., Xin Z., Gan Q., Cheng X., Bartoli F.J. (2013). Plasmonic interferometers for label-free multiplexed sensing. Opt. Express.

[10] Chang Y.-T., Lai Y.-C., Li C.-T., Chen C.-K., Yen T.-J. (2010). A multi-functional plasmonic biosensor. Opt. Express.

[11] Jiang X., Chen Y., Yu F., Tang L., Li M., He J.-J. (2014). High-sensitivity optical biosensor based on cascaded Mach-Zehnder interferometer and ring resonator using Vernier effect. Opt. Lett..

[12] Hsu S.-H., Huang Y.-T. (2005). A Novel Mach-Zehnder Interferometer Based on Dual-ARROW Structures for Sensing Applications. J. Lightw. Technol..

[13] Wu S.Y., Ho H.P., Law W.C., Lin C., Kong S.K. (2004). Highly sensitive differential phase-sensitive surface plasmon resonance biosensor based on the Mach–Zehnder configuration. Opt. Lett..

[14] Tu X., Song J., Liow T.-Y., Park M.K., Yiying J.Q., Kee J.S., Yu M., Lo G.-Q. (2012). Thermal independent Silicon-Nitride slot waveguide biosensor with high sensitivity. Opt. Express.

[15] Zinoviev K.E., González-Guerrero A.B., Domínguez C., Lechuga L.M. (2011). Integrated Bimodal Waveguide Interferometric Biosensor for Label-Free Analysis. J. Lightw. Technol..

[16] Bruck R., Hainberger R. (2014). Sensitivity and design of grating-assisted bimodal interferometers for integrated optical biosensing. Opt. Express.

[17] Liu Q., Kim K.W., Gu Z., Kee J.S., Park M.K. (2014). Single-channel Mach-Zehnder interferometric biochemical sensor based on two-lateral-mode spiral waveguide. Opt. Express.

[18] Ramirez J.C., Lechuga L.M., Gabrielli L.H., Hernandez-Figueroa H.E. (2015). Study of a low-cost trimodal polymer waveguide for interferometric optical biosensors. Opt. Express.

[19] Ma H., Jen A.Y., Dalton L.R. (2002). Polymer-based optical waveguides: Materials, processing, and devices. Adv. Mater..

[20] Hu J., Sun X., Agarwal A., Kimerling L.C. (2009). Design guidelines for optical resonator biochemical sensors. J. Opt. Soc. Am. B.

